# Evolutionary variation of papillomavirus E2 protein and E2 binding sites

**DOI:** 10.1186/1743-422X-8-379

**Published:** 2011-08-01

**Authors:** Adam Rogers, Mackenzie Waltke, Peter C Angeletti

**Affiliations:** 1 Nebraska Center for Virology, School of Biological Sciences, University of Nebraska-Lincoln, Lincoln, NE, 68583-0900, USA

**Keywords:** extrachromosomal DNA, persistent infection, Human papillomavirus, E2 Protein, DNA binding Domain

## Abstract

**Background:**

In an effort to identify the evolutionary changes relevant to E2 function, within and between papillomavirus genera, we evaluated the E2 binding sites (E2BS)s inside the long-control-region (LCR), and throughout the genomes. We identified E2BSs in the six largest genera of papillomaviruses: Alpha, Beta, Gamma, Delta, Lambda, and Xi-papillomaviruses (128 genomes), by comparing the sequences with a model consensus we created from known functional E2BSs (HPV16, HPV18, BPV1). We analyzed the sequence conservation and nucleotide content of the 4-nucleotide spacer within E2BSs. We determined that there is a statistically significant difference in GC content of the four-nucleotide E2BS spacer, between Alpha and Delta-papillomaviruses, as compared to each of the other groups. Additionally, we performed multiple alignments of E2 protein sequences using members of each genus in order to identify evolutionary changes within the E2 protein.

**Results:**

When a phylogenetic tree was generated from E2 amino acid sequences, it was discovered that the alpha-papillomavirus genera segregates into two distinct subgroups (α1 and α2). When these subgroups were individually analyzed, it was determined that the subgroup α1 consensus E2BS favored a spacer of AAAA, whereas subgroup α2 favored the opposite orientation of the same spacer; TTTT. This observation suggests that these conserved inverted linkers could have functional importance.

## Background

Papillomaviruses (PV) are small (55 nm diameter) non-enveloped viruses of icosahedral capsid symmetry that house a single molecule of circular double-stranded DNA [1]. This family of viruses infects surface tissues such as the skin or mucosa which include the mouth, airways, and anogenital tissues of vertebrate animals [2]. Members of the mucosal HPVs are the causative agents of cervical cancer as well as some vaginal, anal, and penile cancers [3-5]. Additionally, emerging research is implicating HPVs in some head and neck cancers [6]. The family of papillomaviridae has 16 assigned genera (alpha-papillomavirus through pi-papillomavirus) and one unassigned genus [7]. There are over 120 strains of HPV identified at present [8] as well as numerous species that infect mammals, birds, and reptiles. Papillomaviruses are classified by differences in the major capsid protein open-reading-frame (ORF), L1. An HPV genotype is defined by a difference of at least 10% in the L1 gene, as compared to the closest known HPV type. A difference of between 2-10% constitutes a subtype, and less than a 2% difference defines a variant [1,9]. Alpha-papillomaviruses are classified into high and low risk categories by their potential to lead to cervical cancer [4,5,10].

The HPV genome that consists of a long control region (LCR), an early gene region, and a late gene set. The LCR (~850 bp) contains the origin of replication (ori) and multiple transcription binding sites, thus controlling the expression of viral genes [1,8]. The compact size of the HPV genome necessitates the use of alternative-splicing for expression of early and late. The early genes are expressed in undifferentiated or newly differentiated keratinocytes, whereas late genes are expressed in keratinocytes undergoing terminal differentiation [1,11]. The early genes (E1, E2, E6 and E7) are primarily responsible for replication, genome maintenance, and the promotion of cell growth. The E2 protein serves as a transcription and replication regulator and a maintenance factor. Full-length E2 protein contains three domains: an N-terminal transactivation domain, an internal "hinge" domain, and a DNA binding domain (DBD) located at the C-terminus. Both the C-terminal and N-terminal domains are relatively well conserved within the PVs [12].

E2 binds as a dimer at DNA-binding sites through the C-terminal DBD [11]. The E2 DBD forms a dimeric^®^-barrel and each strand contributes a half-barrel. The dimer interface has a hydrophobic core and uses extensive hydrogen bonding between subunits to maintain tight binding. This^®^-barrel core contains elaborately packed side chains that contribute to the stability of the dimer, whereas^®^-strands 2 and 3 are connected by a poorly conserved 6-10 residue loop. The tertiary structure of characterized E2 DBDs is similar, but there appear to be variation in the orientations of the two subunits [8]. Some evidence suggests that the activation domain mediates linking activity between E2 molecules bound at distant E2-binding sites, thus forming DNA loops [8,13].

E2 recognizes the consensus sequence, 5'-ACCgNNNNcGGT-3', with nucleotide positions 4 and 9 allowing some variability. A number of studies have examined the binding of E2 protein to its cognate binding site [8,14-20]. The sequence of the 4-nucleotide spacer varies by HPV type, and is thought to be critical for determining E2 binding affinity, and potentially in playing a role in gene regulation, despite having no predicted nucleotide-amino acid contacts from the crystal structure [8,16-18,21]. The E2 homodimer binds the DNA by the alpha helices of each monomer by contact with two successive major grooves of the target site [8,17].

Four E2 binding sites are conserved in the LCR of most papillomaviruses and have been assigned numbers according to their distance from the early promoter [11]. Each site is differentially regulated by variable binding affinity for the E2 protein, resulting in varying replication and transcriptional effects during the viral life cycle [22,23] presumably as a result of differences in E2 binding affinity [8] due to sequence variation as well as methylation of the E2 binding site [14,20]. These binding sites are typically well conserved across all papillomaviruses. However, in some cases variation in the number and location of some E2 binding sites does occur, including a predicted fifth binding site within the LCR of beta-papillomaviruses [24] and some alpha-papillomaviruses [20] as well as observation of up to 17 sequences with ability to bind E2 with the bovine papillomavirus 1 genome [19].

In this study, we examined the evolutionary divergence in E2BS recognition by the E2 transcriptional regulatory protein. Several studies have found that PVs have different numbers of E2BSs with different affinities and different effects on replication [20,25-27]. We hypothesize that PV E2 proteins have evolved different affinities and different preferences for E2BSs, including spacer nucleotides, which control E2BS pre-bend. Currently, the majority of the work performed on the E2 protein function has been performed on domains from a relatively small number of papillomavirus types. A complete understanding of papillomaviruses and the function of their E2 proteins should include all known types. To work towards this objective, we performed a bioinformatic analysis to generate a list of putative E2BS sequences matching the consensus in all papillomaviruses currently classified by ICTV. We then analyzed them for variations in binding site number, location, and differences in the 4-nucleotide spacer region between the largest of the HPV genera, the Alpha, Beta, Gamma, Delta, Lambda, and Xi-papillomaviruses. We performed multiple sequence alignment and phylogenetic analysis of E2 proteins of these viruses to observe evolutionary patterns from an E2-centric perspective. Finally, we performed sequence alignment of the viral E2 protein C-terminal DBDs of each genus and observed that a greater degree of variation is present in the Alpha-papillomaviruses compared to Beta. One of the characteristics associated with the classification of papillomaviruses into their respective genera includes the ability to infect mucosal and cutaneous epithelia as well as fibroblast tissue. Our studies suggest that evolution of the E2 protein and its cognate binding site correlates with adaptive radiation papillomaviruses.

## Methods

### Putative E2 Binding Site Identification and Analysis

Initially, we obtained sequences for the E2 binding sites of three representative, well-characterized papillomavirus species, HPV16, HPV18, and BPV1 [8,28], to create a broad, complete representative training data set. We then utilized Multiple EM Motif Elicitation (MEME) software to use statistic modeling techniques to create a consensus motif sequence for E2 binding sites within the genomes of papillomaviruses [29]. This motif was then used to search through all complete papillomavirus sequences (obtained from the Papillomavirus Episteme (http://pave.niaid.nih.gov/#home, (PaVE)) database containing information from Refseq and Genbank [30-32] for all papillomavirus genera containing 5 of more members (HPV 2-40, 42-45, 47-62, 65-78, 80-96, 99, 100, 102, 104-107, 110, 111, FA75/KI88-03, RTRX7, BPV1-9, COPV, DPV, FdPV1, FdPV2, LrPV1, PlpPV1, PcPV1, UuPV1, and MfPV1-10, utilizing the Motif Alignment and Search Tool (MAST) [33]. For later phylogenetic analyses of alpha-papillomavirus subgroups, we divided our data set to into high and low risk groups and alpha-PVs capable of infecting cutaneous keratinocytes. The high risk group included HPV 16, 18, 26, 31, 33, 35, 39, 45, 52, 56, 58, 59, 67, 73, and 82. The cutaneous subgroup included HPV2, 3, 10, 27, 28, 29, 57, 78, and 94.

### E2BS Sequence Analysis

After retrieving the list of putative E2BSs from the ICTVdb papillomavirus sequences, the data was sorted based on multiple criteria. Recovered sequences were manually analyzed from the resultant MAST output to observe the genome location of the identified binding sites as well as the GC content of the four base spacer sequences. Binding sites were classified as either inside or outside the LCR, according to the criteria of being located between the end of the L1 opening reading frame and the beginning of the E7 open reading frame. Binding sites were similarly separated into their respective papillomavirus genera and the identified E2BSs were analyzed using MEME to generate a Sequence Logo to observe the differences in E2BS consensus sequences for each papillomavirus genus. Similar MEME analysis was performed to compare the E2BSs of low and high-risk alpha-papillomaviruses. Alpha papillomavirus E2BSs were sorted into two subgroups (α1 and α2) based on phylogenetic analyses of E2 proteins (section below). Each of four conserved E2BSs within alpha-HPVs were sorted as to their position within the LCR, for example position 1 E2BSs were compared separately from position 2 E2BSs etc. Analyzed E2BSs were displayed with sequence logo to indicate the extent of conservation at each nucleotide position.

### Protein Sequence Alignment

Amino acid sequences for all known E2 proteins within the papillomaviridae family were acquired from NCBI and sorted into the respective papillomavirus genera analyzed in the previous sections. To refine the significance of our results, analysis was limited to the alpha and beta-papillomavirus genera, as the other genera possess less than ten members each. All E2 sequences were then aligned using Muscle [34]. Some sequences (HPV 77, 3, and 29) were removed due to long stretches of non-homologous repetitive DNA in the linker region. Alignments were then repeated, focusing specifically on aligning the amino acids located within the C-terminal DBD of E2. Weblogo was then used to generate a quantitative graphical representation of the sequence alignments.

### Phylogenetic Analysis

We performed phylogenetic analysis to examine evolution of papillomavirus E2 amino acid sequences. Complete amino acid sequences were obtained from NCBI for all papillomaviruses E2 ORFs and these were subjected to multiple alignment using COBALT software [35]. The multiple alignment was then used to draw phylogenetic trees using Neighbor Joining and Kimura protocols.

## Results

### E2BS Identification

**Figure 1:**
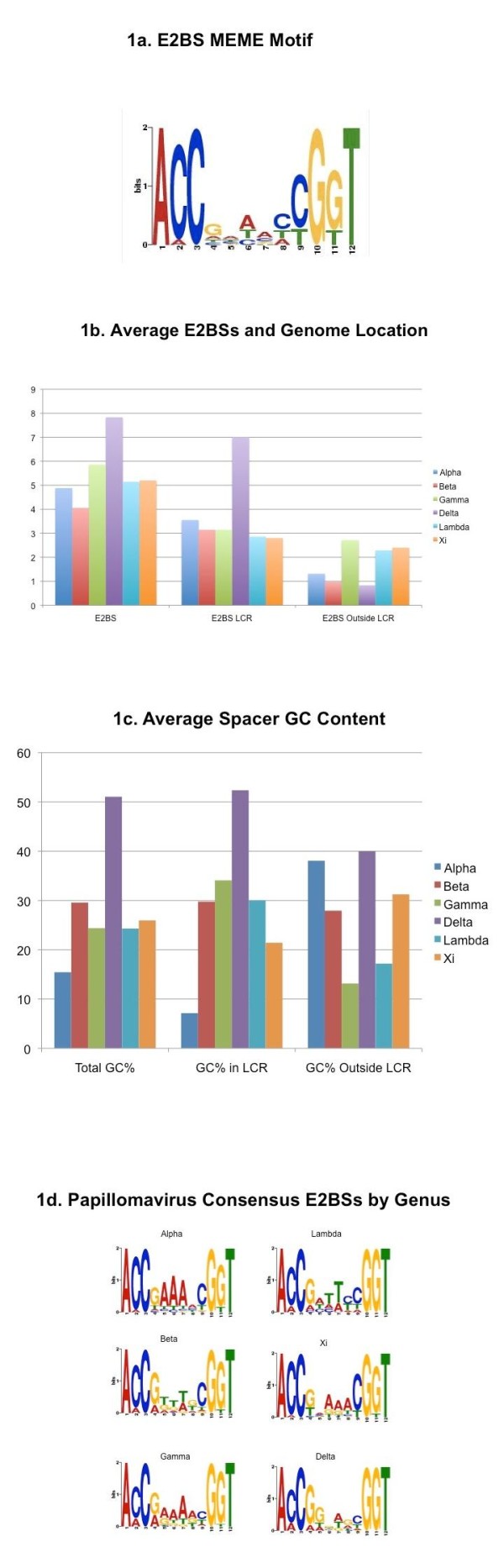
**Consensus Sequence Analysis of E2BSs Throughout Papillomavirus Genera**. Well characterized E2BSs from HPV16, 18, and BPV1 were analyzed using MEME software to generate a consensus E2BS motif **(a)**. This motif was then utilized by MAST software to search through the full-length genomes of 128 papillomaviruses obtained from NCBI to identify sequences with high-identity to the consensus. The average number of E2BSs identified per genome was sorted into the six largest papillomavirus genera and were further analyzed to determine if the binding sites were located inside or outside the LCR of the genomes **(b)**. Identified E2BSs were then manually analyzed to determine the GC content of their four base spacer regions. Results were again calculated in terms of average GC content of E2BSs for each of the individual papillomavirus genera both inside and outside the LCR as well as in total **(c)**. Finally, the identified binding sites were used for MEME analysis to identify the consensus E2BS motif for each of the six papillomavirus genera analyzed in this study **(d)**.

To examine the evolution of E2 DNA binding site sequences, we utilized the sequence motif analysis software MEME to generate a consensus DNA binding site. To generate the initial motif, we generated a training set based on the confirmed E2 binding sites from HPV16 and 18 as well as BPV1, as these are well characterized and representative of the papillomavirus family. The resulting binding site motif is shown in Figure 1a, Sequence Logo, demonstrating the typical high conservation of bases from positions 1-3 and 10-12 along with the lack of sequence conservation in the four base spacer region. Genome sequences were collected from ICTVdb [32] and sorted into the various papillomavirus genera. Papillomavirus genera were eliminated from the rest of the analysis if they contained fewer than five members, in order to improve the statistical significance of results. In total, 68 alpha, 35 beta, 6 delta, 7 gamma, 7 lambda, and 5 xi-papillomaviruses were analyzed, totaling 128 papillomaviruses, representing 111 Human and 17 animal sequences. These were then used to identify the location of E2 binding sites, utilizing MAST software, to identify DNA sequences with high sequence identity to the MEME-generated binding site motif (Figure 1).

As predicted, the four conserved binding sites located within the LCR were identified in the majority of papillomavirus species examined (data not shown). However, a number of potential E2BSs were identified both inside and outside the LCR. The number of E2 binding sites identified averaged between four and six per genome for the alpha, beta, gamma, lambda, and xi-papillomaviruses, whereas the delta-papillomaviruses averaged eight binding sites per genome, (Figure 1b) due in large part to the 14 E2BSs identified in BPV1. The majority of these sequences were found to be located within the LCR as expected, averaging approximately 3 for the alpha, beta, gamma, lambda, and xi, and 7 for delta.

### E2BS Sequence Analysis

The identified E2BSs were then collected and examined to identify the GC content of nucleotides located within their four base spacer regions. G and C nucleotides from the observed E2BSs were counted and tabulated to obtain the average GC content of the four-nucleotide spacer. Most cutaneous papillomavirus genera contained approximately 25 to 30% GC content within the spacer region (Figure 1c). Alpha-papillomaviruses, in general, tended to have very low GC content (15%) and delta-papillomaviruses tended to be very high (approximately 50%, indicating no statistical preference for GC versus AT bases).

When E2BSs were sorted into those "inside the LCR" and "outside the LCR" groups, specific trends became apparent. First, alpha-papillomaviruses and to a lesser extent xi-papillomaviruses displayed a unique requirement for AT nucleotide rich spacers within the LCR, and a much higher GC content in E2BSs located outside. Gamma and lambda-papillomaviruses seemed to possess the opposite trend, with a 15-18% GC content outside the LCR and significantly higher found inside the LCR. Delta-papillomaviruses tended to still have a much higher GC content within the spacer than any of the other papillomavirus genera, while the beta-papillomaviruses remained consistently at approximately 30% GC content.

To further this analysis, we took the identified E2BSs for each papillomavirus genera and performed MEME analysis to identify sequence variation within binding sites by genera (Figure 1d). As predicted, nucleotides 1-3 and 10-12 were well conserved across papillomavirus genera. Some variation was observed in the preference for C and G nucleotides at positions 4 and 9 respectively, particularly in the gamma and delta genera at position 9. The four-nucleotide spacer is highly variable between papillomavirus genera, however some trends are apparent. Alpha-papillomaviruses seemed to have the most consistent sequence conservation, particularly at positions 5-7, in which A nucleotides were very highly conserved. A and T bases were overrepresented in the spacer in all papillomavirus genera, except delta-papillomaviruses, which demonstrated no clear trend for any base at any position. Overall, despite little evidence of evolution of contact nucleotides, we observed that each of the papillomavirus genera seem to have significant variation in preferences for E2BS spacer sequences.

### E2 Protein Phylogenetic Analysis

**Figure 2:**
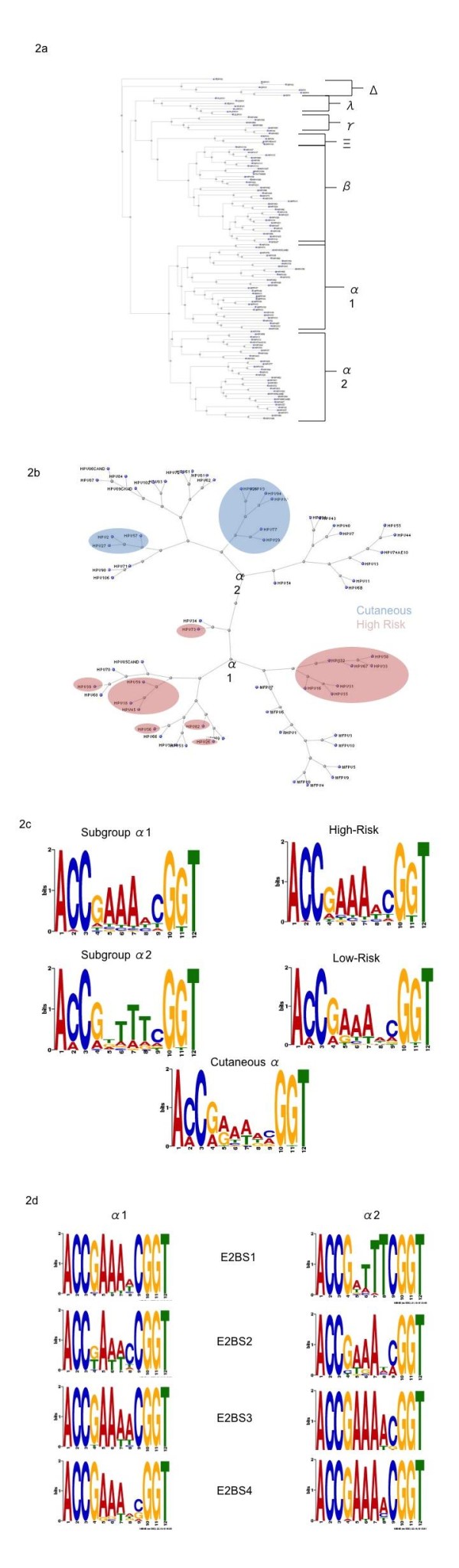
**Phylogenetic Analysis of Papillomavirus E2 Protein and E2BSs**. E2 protein amino acid sequences for each of the papillomaviruses were obtained from NCBI and used for COBALT analysis. The resulting multiple alignment was then used to generate a phylogenetic tree to analyze papillomavirus evolution in terms of the E2 protein **(a)**. Clades were identified corresponding to the classical PV genera and indicated on the tree, as well as two subgroups of the alpha-papillomavirus genera (α1 and α2). These were then expanded and examined individually, and the locations of various types of alpha-papillomaviruses (specifically those capable of infecting cutaneous keratinocytes and those possessing a high-risk of progression to cervical cancer) were indicated **(b)**. HPV E2BSs from part one were then reanalyzed using MEME software to identify a consensus E2BS for each of the subgroups identified in 2b, i.e., subgroup α1 and α2, (high and low-risk alpha-papillomaviruses), as well as those capable of infecting cutaneous keratinocytes tissue **(c)**. Alpha E2BSs of were analyzed for changes in the 4-base pair sequence spacer **(d)**. Each of the four E2 binding sites, numbered 1-4 starting from the closest to the p97 promoter, were analyzed for position-specific differences in the 4-base-pair spacer sequence between alpha subgroups (α1 and α2).

To examine evolution of the E2 protein, we acquired amino acid sequences for all the E2 proteins from papillomaviruses used for the E2BS MEME/MAST procedures. The E2 sequences were then analyzed using COBALT software under Neighbor Joining and Kimura protocols. The resultant phylogenetic tree is shown in Figure 2a. As shown, when analyzed simply from E2 amino acid sequences, papillomaviruses sort into specific clades matching with the genera classifications which, as stated previously, were based on L1 amino acid sequences [7].

Three specific clade groups become apparent based on this analysis: one containing the delta-papillomaviruses, one containing the alpha-papillomaviruses, and a third encompassing the other genera analyzed in this study. The delta clade possessed the largest degree of evolutionary diversity compared to the other clades, implying a significant evolutionary divergence of the delta E2 proteins from the other papillomaviruses. One papillomavirus, FDPV2, did not sort out with the other members of the lambda-papillomavirus genus and, did not associate with any of the other clades identified by this analysis.

The alpha clade further subdivides into two subgroups we labeled as 〈1 and 〈2. When analyzed independently, specific trends become apparent for these two subgroups. The individual members of the subgroups possess specific infectious characteristics (Figure 2b). The majority of the Human papillomaviruses from subgroup 〈1 are associated with the high-risk group of HPVs. One subgroup contains both HPV16 and HPV31, two papillomaviruses most associated with cervical cancer. Interestingly, subgroup 〈1 also contains a cluster of viruses infecting longtailed and rhesus macaques, which seems to have diverged less than the other members of the subgroup in terms of their genetic distance (Figure 2a). Subgroup 〈2 contains two clusters of alpha-papillomaviruses capable of infecting cutaneous keratinocyte cells, as well as three clusters associated with large genital warts (condylomas).

### MEME Analysis of Alpha Subgroup E2BSs

Given the results of the phylogenic analysis for the alpha-papillomavirus genera, we performed MEME analysis on the identified E2BSs for each of the alpha-papillomavirus subgroups, as well as those classified as high and low-risk papillomaviruses and the two clusters containing the alpha-papillomaviruses capable of infecting cutaneous keratinocytes. Given that subgroup α1 consists primarily of high-risk viruses, the consensus motif for subgroup α1 and high-risk alpha-papillomaviruses are essentially identical (Figure 2c). No significant difference was apparent between the high-risk and low-risk viruses outside of a slight under-representation of the guanine nucleotide at position 4, which could suggest a reduced susceptibility at this site for methylation (see discussion). Cutaneous papillomaviruses appear to possess a significantly reduced preference for A/T nucleotides within the four-base-spacer. Interestingly, the subgroup α2 E2BS1, positioned closest to p97, has a consensus motif that has a preference for thymine rather than adenine bases within the four-base spacer (Figure 2d). This would imply that the linker sequence of subgroup α2 is an inversion of the linker from subgroup α1. These differences could be important in the orientation of pre-bending of the E2BS1 DNA in relation to the other E2BSs.

### E2 Amino Acid Sequence Conservation

**Figure 3:**
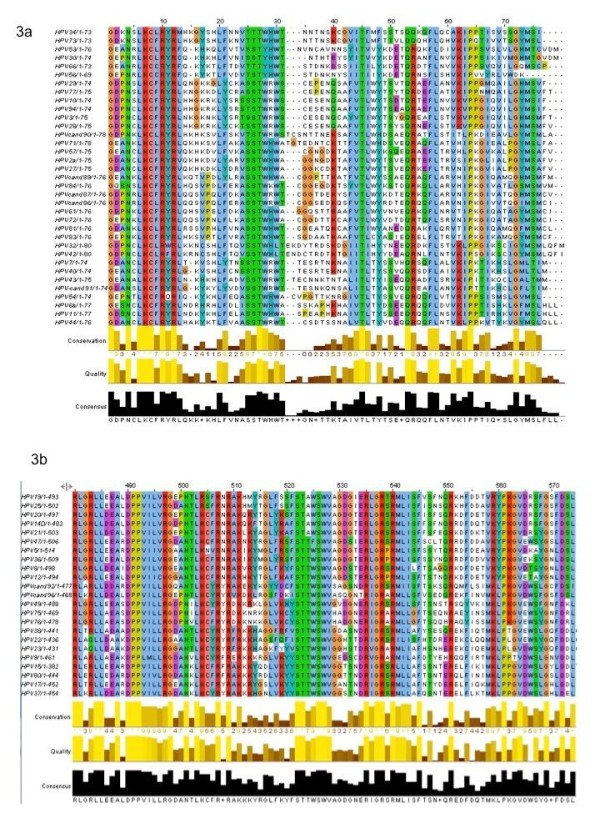
**E2 DNA Binding Domain Protein Alignment**. Amino acid sequences for all known E2 proteins were acquired from NCBI and aligned using Muscle. **(a) **This figure shows the sequence alignment of the Alpha-papillomavirus C-terminal 80 amino acids of the E2 DNA binding domain. The colors represent homologous amino acids and the bar-graphs below represent a quantitative measure of conservation at each position. **(b) **This figure shows the sequence alignment of the Beta-papillomavirus C-terminal 80 amino acids of the E2 DNA binding domain.

**Figure 4:**
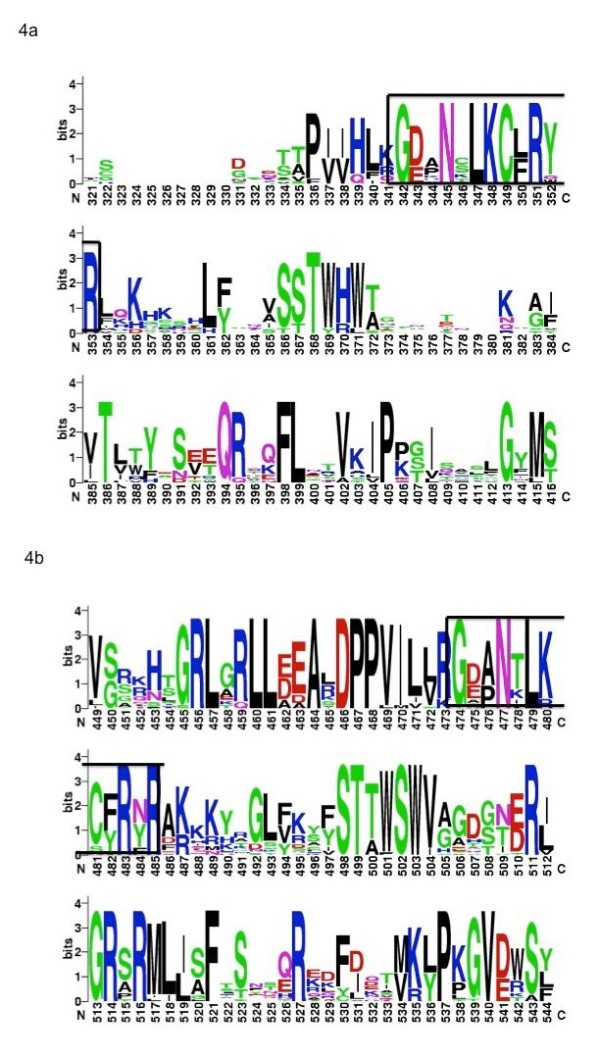
**E2 DNA Binding Domain WebLogo**. Weblogo was used to generate a graphical representation of the sequence analysis of the C-terminal DNA binding domain of E2. The black box represents the conserved region where alpha-papillomavirus E2 proteins contact DNA **(a)**. Similarly, the beta-papillomavirus C-terminal DNA binding domain of E2 alignment is shown. The black box represents the conserved region where alpha-papillomavirus E2 proteins contact DNA **(b)**.

Since one of the primary differences between the alpha-papillomaviruses as compared to the other genera is the ability to infect mucosal, as opposed to cutaneous keratinocytes, we wanted to determine if a similar level of divergence could be observed in the amino acid sequence of the E2 proteins themselves. In order to demonstrate evolutionary divergence of human papillomavirus E2 proteins, complete amino acid sequences for the alpha-papillomaviruses and representative genera of cutaneous papillomaviruses, and the Beta-papillomaviruses were compiled. Certain papillomavirus genera were excluded, since these groups averaged less than ten members each, and thus would make alignments less informative. We initially performed sequence alignments on the full-length E2 protein. However, it was determined that the linker region of Alpha-papillomavirus sequences, which is not well conserved amongst varying HPV types, was skewing the results of the alignments (data not shown). We therefore adjusted our sequences to contain only the C-terminal 80 amino acids of the E2 protein, roughly corresponding to the DNA binding domains (DBD) (Figures 3a, b). It was apparent that alpha-papillomaviruses have a great degree of sequence diversity, as compared to beta-papillomaviruses. A series of representative alignments obtained an average sequence identity of 41% for Alpha-papillomaviruses as compared to 65.25% identity for beta. The differences are also apparent when the logo representative alignment program is used to generate a consensus sequence (Figures 4a, b) even within the, well-conserved region of amino acid sequence, which makes direct contact with the nucleotides of the E2BS.

## Discussion

The vast majority of papillomaviruses analyzed using MEME and MAST during the course of this study conform to the expected number and location of the four conserved E2BSs within the LCRs of their genomes, with some minor variation. The averages across all the genera were between 4-6 E2BSs, besides delta-papillomavirus genus, which seems to be significantly different from the other papillomaviruses. The majority of the sites identified from the study were located within the LCR, though in some cases, sequences that were predicted to bind E2 protein were identified within the papillomavirus ORFs. Whether these putative downstream E2BSs are actually occupied during active infection is an open question, but they could provide a mechanism for regulation of gene expression.

Papillomaviruses are classified by their tissue tropism, genome organization, and sequence divergence within a conserved region of the L1 open reading frame [9]. However, recent phylogenetic analysis has demonstrated that alignment based on the E1 and E2 protein sequences results in a phylogeny which better clusters papillomavirus species in terms of their epidemiology and oncogenicity [28]. The E2 protein is one of four genes which are present in all known papillomaviruses, but has the highest DN/DS ratio of the four, or ratio between non-synonymous versus synonymous substitutions [36]. A DN/DS ratio greater than 1, indicates a high-degree of evolutionary pressure. This is not surprising, since E2 plays numerous functional roles in the cell between regulating transcription, facilitating DNA replication, and viral genome maintenance [11].

E2 proteins bind the consensus palindromic sequence, ACCgNNNNcGGT, through a dynamic, water-mediated interface [8,15]. The NNNN central region or "spacer" is absolutely conserved in length, but the sequence varies by species and individual binding site positions. Hierarchical occupation of the E2BSs by E2 may have important functional and regulatory consequences for both transcription and replication during infection. Previous studies have shown that AT-rich spacers have an increased binding affinity in certain papillomavirus species [8,11]. Specifically, while some alpha-papillomaviruses like HPV16 are acutely sensitive to AT concentration in the spacer region, others like BPV1 are essentially insensitive. Hegde et. al. proposed that the reason for this is due to a reduced ability possessed by the E2 protein of some alpha-papillomaviruses, specifically HPV16, to bend DNA into a conformation which fits within the E2 DNA binding pocket [8]. Essentially, AT-rich stretches of nucleotides are more intrinsically rigid and "pre-bent" into a shape that conforms to the E2 protein DNA binding domain, presumably as a result of binding site-protein co-evolution, thus requiring less energy to deform the target sequence to allow protein binding. The results of this study support this assertion, with alpha-papillomavirus E2BSs possessing approximately 95% A/T nucleotides within the spacer region, as compared to roughly 75% in the cutaneous papillomavirus genera, and 50% in delta-papillomaviruses. With the current limited understanding of nucleotide sequence recognition, specifically for indirect readout which occurs in regions like the E2BS spacer (where no direct nucleotide-amino acid contacts are made), predictions of binding affinity are limited to sophisticated bioinformatic modeling software and empirical data identified using methods like quantitative EMSA. However, regions of increased positive charge tend to correlate favorably with DNA deformation ability, presumably through non-symmetrical charge neutralization by interactions between positively charged amino acid residues and the negatively charged phosphate backbone [37] or by actively attracting the negatively charged DNA to positive residues [38]. Observation of alignments of the Alpha and Beta HPV E2 DNA binding domains (Figure 3, 4) would seem to support this assertion, as a greater number of conserved positively-charged amino acid residues, both within the nucleotide contact region as well as outside, is clearly present in the beta-papillomaviruses. This observation correlates with the increased presence of GC residues in the spacers of Beta-papillomavirus E2BSs. BPV E2 studies have shown that a cluster of positively charged residues located C-terminal of the DBD has been implicated in controlling the sensitivity to the spacer GC content [8]. Interestingly, we observed that the consensus E2BS diverged, even within papillomavirus genera. Specifically, the two alpha subgroups consensus binding site possessed an inverted four base spacer. Typically, when the four conserved binding sites are observed individually, the spacer of binding sites 5' of the viral origin of replication tend to be oriented such that the consensus binding site possesses A nucleotides whereas those 3' of the ori contain the inverse, or T nucleotides [20]. As a result, given that the E2BS sequence is a psuedopalindrome, this would likely result in the E2 protein binding in opposite orientation with respect to the double-helix. The functional consequences of this have yet to be fully explored, but could have interesting implications for E2 function in the two alpha subgroups.

E2BS locations have also diverged along with tissue type, which could have numerous additional effects on viral transcriptional regulation. The number and location of E2BSs varies throughout the PVs. There are 4 primary conserved binding sites near the viral origin of replication termed BS1, BS2, BS3 and BS4. E2 binding to the first site (BS1) interferes with TATA box recognition by the TATA binding protein, binding to the second (BS2) and third (BS3) sites causes promoter repression by competition with cellular transcription factors, and binding to the fourth site (BS4) up regulates viral early gene expression [8]. In addition, binding to BS3 is required for DNA replication. When E2 protein concentration is low, the promoter for the E6 and E7 oncogenes is activated and BS4 is occupied. When E2 protein concentration is high, the E6 promoter is repressed and BS1 and BS2 are occupied by E2 [8]. Differential affinities for the spacers of these E2BSs have been predicted to play a regulatory role in E2 mediated viral gene transcription [8]. The vast differences in number and location of E2BSs identified in this study, however, may suggest that there are significant differences in regulation from one virus species to another. Additionally, the E2 proteins of individual papillomaviruses have demonstrated variable ability to tolerate GC content of the four base spacer [8] and binding site methylation [20] may further individualize the specific regulation strategy utilized.

All four of the E2BSs in the LCR are almost exclusively AT-rich in the spacer. However, predicted E2BSs outside the LCR generally contain higher levels of GC content in the spacer. This suggests that these binding sites would tend to have much lower binding affinity for E2. Considering that external binding sites were not conserved between various HPV types and the fact that E2 has numerous functions that are up or down-regulated during the course of the viral life cycle, it is difficult to speculate what roles these additional binding sites might play, including remodeling the chromosome structure, or potentially blocking the progress of RNA polymerase complexes during transcription. Further complicating the issue is the fact that, in BPV1, 17 total E2 binding sites have been previously identified by gel shift assays, many of which had significantly divergent sequences from the consensus [39]. However, those studies also determined that binding sites more closely related to the consensus generally had the highest binding affinity for E2, thus it is likely that the binding sites identified from this study are preferentially filled at multiple stages of the viral life cycle. This presents a possible regulatory mechanism to control occupation of E2BSs, and thus their transcriptional and/or replicational effects.

One explanation for the greater degree of variability in mucosal HPVs could stem from the wide tissue types infected by Alpha-papillomaviruses. Much of the evolutionary differences observed in the study correlate with differences in preferred infection site. Mucosal epithelia infected by Alpha-papillomaviruses ranges from oral to anogenital, all of which could provide a slightly different micro-environment for HPV replication. Additionally, while cutaneous tissue is considered an immune-privileged site, the mucosal epithelia is much more actively surveyed by the immune system and exposed to IgA. This could also potentially serve as a driving force for divergence of E2 protein function. Previous work has established that differences in tissue type can have significant effect on LCR transcription enhancer activity [40,41]. E2-host co-evolution could then be a potential explanation for the extreme level of tissue specificity exhibited by most members of the papillomaviridae family.

GC content overall tends to be typically low in papillomaviruses, presumably as a means of eliminating targets for methylation by the host gene regulation machinery [20]. Sanchez et. al. determined that there was an evolutionary selection for CpG methylation sites within the E2BSs of papillomaviruses at positions 4-5 and 9-10 [20]. Our analysis demonstrated a varying prevalence of G and C nucleotides, respectively, at these sites between the papillomaviruses. Beta and xi-papillomaviruses, both possessing a much higher prevalence for the CpG methylation site at one or more of the potential sites than the average for the other genera. Delta-papillomaviruses seemed to favor the presence of a methylation site at the 4-5 position, but selected against one at the 9-10 position. For other papillomaviruses, the patterns are somewhat more ambiguous. This is not unexpected, since results by Sanchez et. al. showed that within the alpha-papillomaviruses, the pattern of CpG prevalence varies within the four conserved E2BSs, suggesting that methylation is a key function in determining binding hierarchy for E2 [14,20]. As such, if the same holds true for other papillomavirus genera, it is not surprising that, this pattern would be somewhat skewed. A similar effort to examine the individual conserved E2BSs for papillomaviruses beyond the alpha genus would possibly determine if similar methylation patterns exist, but is beyond the scope of this study.

One important observation from our studies is the large degree of variability between both the proteins and their counterpart DNA binding sites between papillomavirus genera. Delta-papillomaviruses averaged a larger number of E2BSs within the LCR (perhaps, biased somewhat by the 17 E2BSs in BPV1), than any of the other genera examined in this study, and demonstrated a large degree of insensitivity to GC content in the 4-base spacer region. To the other extreme, the alpha-papillomaviruses, showed an intense preference to A/T nucleotides within the four highly-conserved E2BSs in the LCR, almost to the point of exclusion at some base positions. The other genera ranged somewhere in between. It's tempting to infer that, as these three groups primarily infect different tissue types (mucosal epithelia for alpha; cutaneous for beta, gamma, lambda, and xi; and fibroblasts for delta) that this in some way represents an element of the adaptive radiation the virus underwent to adopt these infectious substrates. Aside from potential explanations for this observation, it should remind researchers to be cautious when drawing generalizations between papillomavirus genera E2 proteins, since a particular feature of BPV1 E2 protein may function differently or even be absent for other PVs, as has been shown for HPV16 and BPV1's respective utilization of Brd4 for viral genome maintenance versus regulation of gene expression [42].

## Competing interests

The authors declare that they have no competing interests.

## Authors' contributions

AR performed sequence alignments and analyses and wrote the manuscript. MW did additional data and literature research and helped write the manuscript. PCA conceived of the study and coordinated the work and edited the manuscript.
